# Engineering the holobiont: Synthetic biology strategies for reversing bioenergetic collapse in radiation enteritis

**DOI:** 10.3934/microbiol.2026003

**Published:** 2026-02-13

**Authors:** Yingqi Li, Jiuping Liang, Hui Yin, Tao Liu

**Affiliations:** 1 Shenzhen Bao'an District Songgang People's Hospital, Shenzhen Bao'an Medical College of Guangdong Pharmaceutical University, Shenzhen, 518105, China; 2 Guangdong Provincial Key Laboratory of Pharmaceutical Bioactive Substances, School of Basic Medical Sciences, Guangdong Pharmaceutical University, Guangzhou 510006, China

**Keywords:** radiation enteritis, microbial synthetic biology, holobiont engineering, immunomodulation, bioenergetic collapse, living biotherapeutic products, *Akkermansia muciniphila*, colonic terraformation, logic gates, gut-on-a-chip

## Abstract

Radiation enteritis (RE) constitutes a catastrophic collapse of the intestinal holobiont, fundamentally constraining the curative potential of abdominopelvic radiotherapy. Current clinical management, limited by a reductionist focus on host DNA damage, often overlooks the maladaptive ecological feedback loops driving chronic pathology. This review advocates for a definitive transition from empirical probiotic supplementation to microbial synthetic biology for the rational reprogramming of the gut ecosystem. We delineate strategies for thermodynamic engineering, utilizing engineered commensals as living oxygen sinks to scavenge luminal oxygen and nitrate, thereby starving proteobacterial blooms and restoring the physiological hypoxia essential for mucosal recovery. Addressing the *Akkermansia* paradox, in which nutrient deprivation drives beneficial microbes toward mucin-degrading pathogenicity, we propose genetic domestication (e.g., sulfatase deletion) to decouple immunogenicity from barrier erosion. Furthermore, we explore the design of biological computers equipped with Boolean logic gates to resolve the immunological timing paradox by precisely modulating the cGAS-STING axis and reprogramming macrophage metabolism via the itaconate shunt. Integrating armored polydopamine delivery, genetic entanglement (STALEMATE) for biocontainment, and Gut-on-a-Chip validation, we outline a roadmap for colonic terraformation. This engineering-driven approach aims to actively reconstruct homeostasis, uniquely decoupling epithelial regeneration from tumor protection to improve long-term cancer survival.

## 1. Introduction

**Figure 1.**
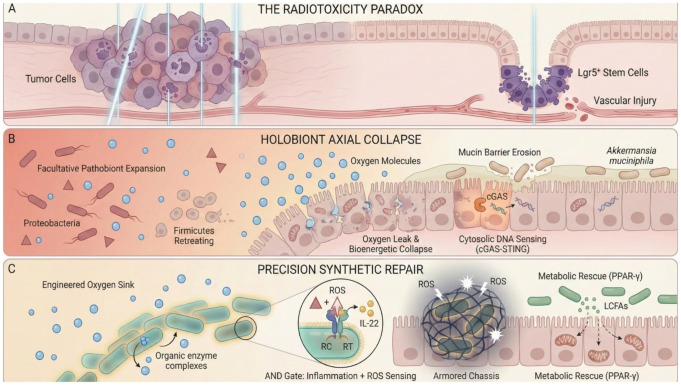
Schematic representation of the transition from bioenergetic collapse to precision synthetic reconstruction. (A) The radiotoxicity paradox: radiotherapy effectively targets tumor cells but simultaneously devastates the *Lgr5*^+^ intestinal stem cell niche and causes vascular injury, initiating the pathological cascade. (B) Holobiont axial collapse: Radiation-induced mitochondrial dysfunction leads to an oxygen leak (blue spheres) into the lumen. This thermodynamic shift drives the expansion of facultative proteobacteria and the retreat of obligate firmicutes. Under starvation pressure, *A. muciniphila* shifts to a mucin-degrading pathobiont phenotype, eroding the barrier, while cytosolic DNA activates the cGAS-STING inflammatory axis. (C) Precision synthetic repair: Strategies for colonic terraformation. Engineered commensals act as oxygen sinks to restore hypoxia. Armored chassis protect therapeutic strains, while logic gates, specifically AND gates, sense inflammation and ROS to precisely release regenerative factors, e.g., interleukin-22 (IL-22), or metabolic signals (LCFAs) to reignite host mitochondrial β-oxidation.

Abdominopelvic radiotherapy remains a cornerstone of oncological care, yet its curative potential is fundamentally constrained by the radiotoxicity paradox [1],[2] (Figure 1A). While ionizing radiation effectively induces lethal DNA breaks in malignant tissues, it simultaneously devastates the rapidly proliferating intestinal epithelium and manifests as radiation enteritis (RE) in up to 70% of patients [3],[4]. Historically, the clinical understanding of RE has been dominated by the reductionist target theory [5],[6]. This host-centric model attributes tissue failure primarily to stochastic DNA damage within the *Lgr5*^+^ stem cell niche. However, this perspective is increasingly insufficient as it fails to account for the complex luminal microenvironment. Consequently, standard interventions often prove maladaptive as they fail to account for the spatial heterogeneity of the gut [7]; specifically, the differential oxygen gradients between the small intestine and colon necessitate distinct therapeutic targets [8]. The oxygen hypothesis, therefore, must be refined to account for these physiological variations. Notably, the prophylactic use of broad-spectrum antibiotics frequently exacerbates pathology by decimating the commensal anaerobic guild essential for colonization resistance [9],[10].

Emerging evidence necessitates a paradigm shift toward a holobiont perspective that treats the host and its resident microbiota as a singular, co-evolved biological unit [9]. Within this framework, RE represents not merely a tissue injury but a catastrophic collapse of the gut microbiota–immune–metabolism axis [11] (Figure 1B). Central to this failure is the disruption of the gut's bioenergetic economy. Under physiological conditions, specific commensals such as *Holdemanella biformis* drive epithelial mitochondrial β-oxidation through peroxisome proliferator-activated receptor-gamma (PPAR-γ) signaling [12]. This high-rate metabolism actively consumes oxygen to maintain a steep radial oxygen gradient known as the biological oxygen barrier. The combined insults of direct mitochondrial damage and the depletion of butyrate-producing anaerobes force the epithelium into a Warburg-like glycolytic shift. Since glycolysis does not consume oxygen, the biological barrier fails and results in an oxygen leak where oxygen freely diffuses into the lumen. This influx alters the thermodynamic landscape, though with distinct regional dynamics. Radiation-induced vascular injury exacerbates this by disrupting the capillary–epithelial barrier, allowing oxygen from hyperpermeable vessels to diffuse into the lumen. This paradox of deep tissue ischemia and luminal hyperoxia fuels a predictable bloom of facultative proteobacteria and aerotolerant firmicutes (e.g., *Enterococcus*) at the expense of obligate anaerobes [8],[13]. This phenomenon, formalized as the oxygen hypothesis [9], suggests that dysbiosis is a symptom of an underlying thermodynamic failure rather than a stochastic ecological drift.

Furthermore, radiation enteritis constitutes an evolutionary mismatch where ancient repair mechanisms become maladaptive in the face of the modern evolutionary novelty of high-energy ionizing radiation [14],[15]. The cyclic GMP-AMP synthase (cGAS) stimulator of interferon genes (STING) pathway, originally evolved to sense viral pathogens, is hijacked by radiation-induced micronuclei to drive a sterile and chronic type-I interferon response that impairs regeneration [11]. This context-dependent maladaptation extends to the microbiota itself, best exemplified by the *Akkermansia* paradox. While typically a guardian of barrier integrity, *Akkermansia muciniphila* switches to an aggressive mucin-degrading pathobiont phenotype within the nutrient-depleted environment of the irradiated gut. This necessitates a shift in therapeutic strategy from empirical supplementation to rational ecological engineering.

Given that RE represents a systemic failure of the regulatory circuits within the holobiont, empirical supplementation with wild-type probiotics is increasingly insufficient [16]. Such rudimentary interventions fail to address the complexity of the dysbiosis and the distinct thermodynamic constraints of the injured gut. Consequently, this review advocates for a definitive transition from a descriptive mechanism-driven understanding to a prescriptive engineering-driven approach utilizing microbial synthetic biology (Figure 1C) [17],[18]. By viewing pathological features such as luminal hyperoxia, immune hyperactivation, and metabolite depletion as specific design constraints, commensal bacteria can be reprogrammed to function as precision therapeutic machines. Here, we delineate a roadmap for colonic terraformation—explicitly defined as the active reconstruction of gut metabolic, immunological, and ecological homeostasis via synthetic biology. This engineering-driven approach integrates thermodynamic engineering, logic-gated immunomodulation, and metabolic rescue strategies to actively reverse the collapse of the holobiont.

## 2. Thermodynamic engineering strategies for hypoxia restoration

Radiation-induced dysbiosis manifests as a proteobacterial bloom defined by the expansion of facultative anaerobes like Enterobacteriaceae at the expense of the obligate anaerobic guilds [19]. The driving force is a disruption of the bioenergetic economy where the availability of high-energy electron acceptors redefines the competitive hierarchy [20]. Reversing this state requires a transition from empirical probiotic supplementation to thermodynamic engineering. This approach involves the rational design of microbial chassis capable of scavenging electron acceptors and restoring the reductive potential necessary for mucosal homeostasis [21].

### 2.1. Mechanisms of bioenergetic collapse and the oxygen hypothesis

The mechanistic basis for the pathogen bloom is articulated by the oxygen hypothesis [9]. In the healthy intestine, a steep radial oxygen gradient is actively maintained by the host epithelium [8]. Functional colonocytes act as a biological oxygen barrier by engaging in high-rate mitochondrial β-oxidation of microbiota-derived short-chain fatty acids [19]. This metabolic configuration consumes diffused oxygen and maintains luminal hypoxia below 1% saturation, which is a niche requirement for the dominant obligate anaerobes lacking catalase and superoxide dismutase enzymes [22].

We propose a tripartite bioenergetic collapse model synthesized from recent findings on mitochondrial damage and metabolic reprogramming [23]–[25]. First, ionizing radiation directly compromises mitochondrial DNA and disrupts electron transport chain complexes in epithelial cells [23]. Second, the depletion of butyrate-producing Clostridia starves colonocytes of their primary energetic substrate [26]. Third, surviving colonocytes undergo a Warburg-like metabolic shift from oxygen-consuming β-oxidation to anaerobic glycolysis to generate adenosine triphosphate (ATP) [24]. Oxygen diffuses from the submucosal capillary bed through the epithelium and into the intestinal lumen, creating an oxygen leak [13]. Crucially, this leak is not uniform; radiation-induced vascular damage (endarteritis) compromises the diffusion barrier, allowing oxygen from the submucosal plexus to flood the lumen [27],[28]. This highlights that re-establishing hypoxia requires not just epithelial repair but the active scavenging of vascular-derived oxygen [19]. This influx acts as a selective toxin to obligate anaerobes while providing a high-energy electron acceptor for facultative proteobacteria [13],[28]. Thermodynamically, aerobic respiration yields approximately 15 times more ATP per mole of substrate than fermentation, granting these pathobionts a substantial growth advantage [9].

### 2.2. Inflammatory electron acceptors as drivers of pathogen expansion

The dysbiotic shift is exacerbated by the generation of alternative electron acceptors derived from the host inflammatory response. Radiation-induced reactive oxygen species (ROS) react with nitric oxide to form peroxynitrite, which degrades into nitrate. Concurrently, the oxidation of luminal thiosulfate by inflammatory ROS generates tetrathionate [26]. Unlike fermentation-reliant anaerobes, Enterobacteriaceae encode specific respiratory reductases, allowing them to use these molecules as terminal electron acceptors [29],[30]. This nitrate respiration effectively electrifies the gut lumen and creates a high-energy metabolic niche that fuels the pathobiont bloom in a self-reinforcing cycle [28]. These electron acceptors drive a strongly directional ecological shift favoring proteobacteria dominance.

### 2.3. Rational design of living oxygen scavengers and metabolic rescue

Restoring the anaerobic status of the gut is a fundamental prerequisite for resolving radiation enteritis (RE). A multi-layered synthetic biology approach reverses this thermodynamic catastrophe by engineering the restoration of the anaerobic niche [28].

The first design strategy focuses on the oxygen scavenging chassis. To compensate for the loss of epithelial oxygen consumption, *Escherichia coli* Nissle 1917 (EcN) can be engineered to function as a living oxygen sink. This strain naturally encodes cytochrome *bd* oxidase, which is a high-affinity terminal oxidase capable of functioning under microaerophilic conditions [31]. Placing the *cydAB* operon under the control of an oxygen-sensitive promoter like fumarate and nitrate reduction regulator (FNR) allows the engineered strain to overexpress oxygen scavenging machinery specifically in the microaerophilic zones of the irradiated gut [32]. This engineered strain acts as a metabolic shield, aggressively consuming oxygen to re-establish the hypoxic gradient [33]. However, relying solely on this cooperative function introduces a risk of ecological fragility [34]. Ecological network modeling indicates that microbial communities dominated by strong cooperative interactions are susceptible to instability, whereas competitive interactions dampen positive feedback loops and promote stability [35]. Therefore, the engineered scavenger must be viewed as a transitional pioneer; long-term stability requires the re-establishment of a competitive anaerobic guild that dampens these loops and ensures redundancy in oxygen consumption [36],[37].

The second strategy involves a tetrathionate decoy to starve proteobacteria of inflammatory electron acceptors. An engineered strain overexpressing a high-efficiency tetrathionate reductase system, such as *ttrBCA*, but lacking downstream virulence pathways, competes with pathogens like *Salmonella* for tetrathionate substrates. By reducing tetrathionate to benign thiosulfate without triggering inflammation, the engineered strain deprives pathobionts of their thermodynamic fuel via exploitative competition [18].

A definitive resolution requires metabolic augmentation to reactivate the host oxygen consumption machinery. Recent breakthrough findings identify *Holdemanella biformis* as a critical factor in restoring hypoxia via host signaling. *H. biformis* synthesizes specific long-chain fatty acids such as 3-hydroxyoctadecaenoic acid, which function as ligands for PPAR-γ on the host epithelium [12]. Activation of PPAR-γ upregulates genes encoding mitochondrial β-oxidation enzymes, thereby restarting host oxygen consumption and closing the oxygen leak. As *H. biformis* is often depleted in RE patients, a synthetic biology approach involves cloning the LCFA biosynthetic gene clusters, including oleate hydratase, into a robust delivery vector like *Limosilactobacillus reuteri*. This heterologous expression system delivers the metabolic signal to re-ignite epithelial mitochondria, restoring the epithelial bioenergetics to reshape the microbiota to favor the recovery of a healthy fermentative anaerobic guild [24].

Beyond hypoxia restoration, correcting the metabolic imbalance requires targeting alternative electron acceptors, such as nitrate, and the active supply of energetically costly metabolites to fuel mucosal regeneration. For instance, addressing the nucleotide starvation inherent to radiation-induced mitotic death or deploying eukaryotic chassis to bypass antibiotic-associated dysbiosis represents the next frontier of holobiont engineering. Representative examples of these thermodynamic and metabolic engineering modules, including novel eukaryotic and nucleotide-secreting platforms, are detailed in Table 1.

While thermodynamic engineering effectively addresses the abiotic constraints of the irradiated gut by restoring hypoxia, it fails to resolve the context-dependent pathogenicity of the microbiota [38]. Lowering redox potential is a prerequisite for recovery, but it does not inherently prevent nutrient-deprived commensals from turning parasitic [39]. This limitation necessitates a shift to ecological engineering, specifically designed to resolve maladaptive behaviors such as the *Akkermansia* paradox [40].

**Table 1.** Engineered microbial modules for thermodynamic calibration and metabolic rescue.

Target mechanism	Microbial chassis	Key genetic modification	Therapeutic outcome	Ref
Hypoxia restoration	EcN	VHb (high-affinity hemoglobin)	Consumes leaked oxygen to restore the anaerobic niche.	[41]
Redox lowering	*Lactococcus lactis*	NoxE (NADH oxidase)	Rapidly converts O₂ into H_2_O, protecting anaerobes.	[42]
Pathogen starvation	*E. coli* (commensal)	NapABC (nitrate reductase)	Scavenges nitrate to starve *Salmonella*/*E. coli*.	[43]
Metabolic support	*S. cerevisiae* (yeast)	Butyrate pathway (*ERG10-ter*)	Delivers butyrate energy directly to colon cells.	[44]
Barrier repair	*Bacillus subtilis*	BCoAT + Δ*ackA*	Spore-based delivery of butyrate for mucosal healing.	[45]
Wound healing	*E. coli* mutant	Δ*purR* (purine deregulation)	Secretes purines (ATP precursors) to fuel regeneration.	[46]

Abbreviations: EcN, *Escherichia coli* Nissle 1917 (probiotic chassis); VHb, *Vitreoscilla* hemoglobin (enhances microaerophilic respiration); NoxE, H₂O-forming NADH oxidase (lowers redox potential without ROS generation); NapABC, periplasmic nitrate reductase (nitrate sink); ERG10-ter, heterologous butyrate biosynthetic pathway; BCoAT, butyryl-CoA:acetate CoA-transferase; ΔackA, acetate kinase deletion (blocks acetate overflow); ΔpurR, purine repressor deletion (constitutive purine secretion).

## 3. Ecological engineering to resolve the *A. muciniphila* paradox

The radiation-induced collapse of the gut ecosystem extends beyond thermodynamics to a profound nutritional crisis that redefines host–microbe interactions [47]. While homeostatic mutualism relies on the host providing dietary fibers and secreted mucins to sustain its symbionts, radiation abruptly severs these nutrient supply chains [48]. This starvation pressure triggers a survivalist switch in specific commensals, forcing them to transition from mutualistic guardians to opportunistic pathobionts [49]. Nowhere is this ecological inversion more clinically critical than in the behavior of the mucin-specialist *A. muciniphila*, a paradox that necessitates the design of rational synthetic biology strategies to engineer the interaction from parasitism back to commensalism.

### 3.1. Context-dependent transition from commensalism to pathogenicity

In the healthy microbiome, *A. muciniphila* is widely hailed as a next-generation probiotic and sentinel of barrier integrity [50],[51]. Residing in the outer mucus layer, it constitutively degrades mucin glycans, a process that under homeostatic conditions stimulates the host to replenish the mucus barrier, thereby maintaining thickness and viscosity [49]. Furthermore, *A. muciniphila* secretes the outer membrane protein Amuc_1100, which acts as a molecular ligand for Toll-like receptor 2 (TLR2) on intestinal epithelial cells. This signaling cascade reinforces the paracellular barrier by upregulating tight junction proteins such as Claudin-3 and Occludin and inducing anti-inflammatory interleukin-10 secretion [52]–[54].

However, recent mechanistic insights have challenged this monolithic view, unveiling a functional duality driven by the environmental context. A pivotal study provided definitive evidence that in the specific context of acute radiation injury, *A. muciniphila* switches to an aggressive pathogenic phenotype [11]. The driver of this transition is nutritional starvation. Ionizing radiation decimates the intestinal stem cell pool and secretory goblet cells responsible for mucin production [47]. Concurrently, patients often experience anorexia or restricted diets, leading to fiber deprivation. In this mucin-depleted environment, *A. muciniphila* faces existential metabolic stress. Unlike generalist *Bacteroides* species that switch between dietary polysaccharides and host glycans, *A. muciniphila* is a mucin specialist [48]. Transcriptomic analysis of *A. muciniphila* isolated from irradiated murine guts reveals a dramatic upregulation of the mucin utilization locus. This arsenal includes glycosyl hydrolases and, critically, S1 family sulfatases [55],[56]. Mucin glycans in the distal colon are heavily sulfated to protect against rapid bacterial degradation. Sulfatases act as the key to the lock by cleaving terminal sulfate residues before other hydrolases can access the glycan core [57]. The upregulation of these enzymes allows the bacterium to aggressively harvest remaining host glycans, effectively eating the house that protects the host. This enzymatic stripping breaches the compromised barrier, allowing pathogen contact with the epithelium. This translocation triggers macrophage NLRP3 inflammasome activation, releasing IL-1β and IL-6, which block Wnt signaling and inhibit goblet cell regeneration [11]. Thus, a vicious cycle is established where radiation-induced mucin depletion drives *Akkermansia* virulence, which further erodes the barrier and blocks repair. This pathobiont bloom significantly exacerbates mortality in radiation models, underscoring the risks of indiscriminate probiotic supplementation in oncology [58].

### 3.2. Genetic domestication via targeted sulfatase deletion

A theoretical engineering strategy involves creating a domesticated *Akkermansia* strain via precision genomic editing. Although currently a forward-looking concept requiring *in vivo* validation in the context of RE [59], recent breakthroughs in the genetic manipulation of verrucomicrobia, including transposon mutagenesis and modular shuttle vectors, have rendered *A. muciniphila* genetically tractable [60], making this domestication concept technically feasible. This approach aims to decouple immunogenicity from barrier erosion. The objective is to retain expression of the surface pili (Amuc_1100) necessary for TLR2-mediated barrier strengthening while genetically disabling the enzymatic machinery required for deep mucin erosion [11].

Utilizing these tools, defined knockout strains deficient in S1 family sulfatases can be engineered. This engineered *Akkermansia* would be unable to initiate degradation of sulfated mucins found in the deep inner mucus layer, effectively locking it out of the host's primary defense barrier [57]. However, it would retain the ability to graze on non-sulfated outer mucus or rely on metabolic cross-feeding from other commensals. In the context of radiation, this domesticated strain provides TLR2 stimulation to promote epithelial healing without possessing the enzymatic capacity to strip the protective barrier, effectively converting a conditional pathobiont into an obligate commensal [60]. We summarize selected advanced engineering architectures for managing the *Akkermansia* population and preserving the mucin barrier in Table 2.

**Table 2.** Synthetic strategies for modulating mucin ecology and restoring barrier integrity.

Strategy tier	Synthetic platform	Mechanism	Therapeutic rationale	Ref.
Domestication	*A. muciniphila* Δ*sul* / Δ*GH*	Deletes sulfatases/sialidases	Prevents deep mucin degradation while retaining Amuc_1100 signaling.	[60]
Spatiotemporal	*E. coli* Nissle Δ*alr* Δ*dadX*	D-Ala auxotrophy	Prevents systemic spread (lysis without D-Ala).	[46]
Spatiotemporal	*L. lactis* Δ*thyA*	Thymidine auxotrophy	Clinical-grade containment; cell death without thymidine.	[61]
Spatiotemporal	*E. coli* GRO (C321.ΔA)	Synthetic auxotrophy (BipA)	Prevents evolutionary escape via genetic recoding.	[62]
Spatiotemporal	*E. coli* Nissle TSMs	Thermosensitive meganucleases	Self-destruction < 22 °C.	[63]
Decoy	*E. coli* Nissle 2′-FL+	Secretes 2′-Fucosyllactose	Distracts mucin foragers with soluble decoys.	[64]
Decoy	*E. coli* PATCH	Secretes CsgA-TFF3 fibers	Self-assembling wound sealant; biomimetic barrier.	[65]
Decoy	*L. lactis* Surface-Amuc	Displays Amuc_1100 protein	Safe TLR2 barrier activation without live *Akkermansia*.	[66]
Decoy	*L. lactis* P9	Secretes GLP-1 inducer P9	Systemic metabolic repair and epithelial restitution.	[67]

Abbreviations: Δsul / ΔGH, deletion of sulfatase and glycosyl hydrolase genes (“genetic domestication”); Δalr/ΔdadX, alanine racemase/dehydrogenase deletion; ΔthyA, thymidylate synthase deletion; Amuc_1100, *A. muciniphila* outer membrane protein (TLR2 ligand); TSMs, thermosensitive meganucleases (biocontainment < 22 °C); GRO, genomically recoded organism; BipA, synthetic L-4,4′-biphenylalanine (auxotrophy enforcement); 2′-FL, 2′-fucosyllactose (soluble mucin decoy); PATCH, probiotic-associated therapeutic curli hybrids (CsgA-TFF3 matrix); P9, *A. muciniphila* secreted protein (GLP-1 inducer).

### 3.3. Material-driven decoy strategies for mucin barrier preservation

An alternative strategy employs material-driven engineering to protect the mucus barrier via metabolic interference. This approach utilizes decoy strategies to divert *Akkermansia* foraging activity away from the host epithelium (Table 2).

Advances in polymer chemistry have enabled the synthesis of mucin-mimetic polymers, via ring-opening metathesis polymerization, that structurally replicate the mucin backbone and display high-density glycan ligands such as N-acetylgalactosamine [68]. By orally delivering these synthetic mucins, the gut lumen is flooded with high-affinity decoy substrates [69]. The engineered polymers act as a molecular sponge, attracting and binding *Akkermansia* via its mucin-binding domains [70]. This effectively saturates the bacterium's enzymatic machinery, directing its degradative activity toward the sacrificial decoy rather than the fragile host barrier. Crucially, this preserves the physical integrity of the host mucus while maintaining the presence of *Akkermansia* for its immunological benefits. This strategy creates a metabolic shield that allows the host epithelium to regenerate under the cover of a synthetic sacrificial layer, breaking the cycle of inflammation and erosion [71],[72].

## 4. Programmable logic circuits for spatiotemporal immunomodulation

While ecological engineering restores the players, it does not inherently control their timing or spatial restriction. To resolve the immunological timing paradox and prevent off-target effects in residual tumors, we must deploy biological computers—commensals equipped with programmable logic circuits capable of autonomous decision-making.

The immunological response to abdominopelvic radiotherapy is defined by a critical timing paradox wherein the molecular drivers of regeneration can transition into the engines of fibrosis [73]. While transient acute inflammation is a prerequisite for crypt fission and epithelial restitution, the persistence of these signals drives chronic pathology and stricture formation [74]. Standard pharmacological interventions, which typically act as monotonic suppressors, lack the temporal resolution to navigate this complexity [75]. Current synthetic approaches address this by utilizing simple sensing circuits; however, the application of complex Boolean logic gates for RE represents a novel, forward-looking engineering frontier [76]–[78].

### 4.1. Temporal regulation of the cGAS-STING axis using genetic toggle switches

The cytosolic DNA-sensing pathway, cGAS-STING, exemplifies the evolutionary mismatch central to RE [79],[80]. During the 0–96 h acute phase, radiation-induced cytosolic DNA triggers a cGAS-dependent type-I interferon pulse that functions not merely as a toxin but also as an essential priming cue for *Lgr5*^+^ intestinal stem cells [81],[82]. Without this signal, compensatory proliferation fails [83]. However, the failure to clear radiation-induced micronuclei leads to constitutive STING activation in the chronic phase, which hijacks the non-canonical PERK-eIF2α axis to drive the senescence-associated secretory phenotype and fibrosis [84],[85].

To resolve this paradox, we propose engineering EcN with a genetic toggle switch functioning as a temporal differentiator. This circuit architecture utilizes a bistable genetic memory element coupled to a quorum-sensing timer. Upon initial colonization, representing the acute state, the bacteria constitutively express a STING agonist, such as cyclic di-AMP, to amplify the host's innate regenerative pulse and ensure robust barrier restitution [82],[86]. As the bacterial population density reaches a threshold calibrated to the transition to chronicity, the accumulation of autoinducers triggers the toggle to flip. This state transition represses the agonist and activates the secretion of DNase and ENPP1 [87]. These enzymes degrade luminal cell-free DNA and cGAMP, effectively scrubbing the inflammatory triggers to terminate signaling and prevent fibrotic remodeling [84].

### 4.2. Combinatorial logic gates to decouple regeneration from tumor protection

To strictly confine therapeutic efficacy to the injured intestine and avoid shielding residual tumor cells (the radiotoxicity paradox), we propose the integration of an AND logic gate [88]. This design exploits tetrathionate as a specific biomarker of luminal inflammation [89]. It is important to note that the tumor microenvironment (TME) is not strictly reducing; rather, it is often characterized by pro-oxidative stress driven by metabolic dysregulation [90]. Therefore, a simple ROS sensor might inadvertently trigger in the tumor. To rigorously prevent this, the circuit utilizes a combinatorial AND gate: requiring both luminal tetrathionate (specific to inflammation) AND NO lactate (tumor signature), thereby creating a combinatorial logic gate with signal inversion that strictly confines regenerative payloads to the non-malignant mucosa [9],[88]. To achieve this, the engineered circuit couples the tetrathionate sensor (*ttrS/R*) with a lactate-repressible promoter repressible by high concentrations of lactate, ensuring therapeutic payload release occurs only in the inflamed (tetrathionate-rich) but non-malignant (lactate-low) niche [91],[92]. The therapeutic output, such as IL-22, is placed under the control of a combinatorial promoter that requires the presence of tetrathionate and the absence of lactate to trigger therapeutic release. Consequently, the bacterium functions as a micro-physician that dispenses regenerative factors only when it detects the specific chemical signature of radiation colitis, remaining inert within the tumor core.

Beyond combinatorial logic, the precision of microbial immunotherapy can be further refined by deploying modular biosensors tuned to the specific chemical signatures of the radiation-injured microenvironment. These biomarker-responsive actuators exploit evolutionarily conserved sensing domains to autonomously diagnose local pathology. Representative examples of these advanced sensing architectures are provided in Table 3.

**Table 3.** Genetic circuit architectures for spatiotemporal immunomodulation and sensing.

Strategy	Genetic sensor/regulator	Output payload	Mechanism of action in the holobiont	Ref.
Nitric oxide sensing	NorR + PnorVβ	Anti-TNFα VHH	Neutralizes TNFα (KD ≈ nM) in high-NO zones; halts apoptosis.	[93]
Zinc sensing	Zur + PykgMO	Interleukin-10	Senses Zn²⁺ depletion; IL-10 resolves inflammation/DAI.	[94]
Quorum lysis	LuxI/R + φX174E	Anti-CD47 VHH	Cyclic lysis releases VHH; blocks CD47 to boost efferocytosis.	[95]
Nitrate Respiration	NarX-NarL + PyeaR	Indole-3-Acetic Acid	Senses NO₃⁻; IAA activates AhR/IL-22 to repair barrier.	[96]
Thermal switch	TlpA/TcI + PtlpA/R	Toxin/Kill Switch	FUS-triggered (>42 °C) local release; precise spatial control.	[97]

Abbreviations: NorR, NO-sensing transcriptional regulator; VHH, variable heavy chain homodimer (nanobody); Zur, zinc uptake regulator (senses zinc depletion); LuxI/R, quorum-sensing synthase/regulator; φX174E, bacteriophage lysis gene E (inhibits MraY); NarX-NarL, nitrate/nitrite two-component sensor system; TlpA, *Salmonella* thermosensitive repressor (unfolds >42 °C); TcI, temperature-sensitive λ cI repressor.

### 4.3. Macrophage reprogramming via the engineered itaconate shunt

Beyond signal transduction, chronic inflammation is sustained by a metabolic deadlock in resident macrophages. Radiation injury forces lamina propria macrophages into a glycolytic M1 phenotype characterized by a break in the Krebs cycle at succinate dehydrogenase (SDH). This leads to succinate accumulation, which stabilizes hypoxia-inducible factor-1α (HIF-1α) and drives sustained IL-1β secretion [98],[99].

To break this cycle, we propose engineering a metabolic shunt within the microbiome. A paradigmatic strategy involves introducing the *cis*-aconitate decarboxylase gene, *cadA*, into the probiotic chassis; the bacterium diverts its TCA cycle flux to synthesize itaconate. This immunometabolite is secreted into the crypt microenvironment, where it is taken up by host macrophages. Intracellular itaconate acts as a competitive inhibitor of SDH and an alkylator of Kelch-like ECH-associated protein 1, activating the Nrf2 antioxidant response. This exogenous metabolic intervention bypasses the host's enzymatic deficiencies, effectively forcing macrophages out of the pro-inflammatory M1 state and into a restorative M2 phenotype [100]. By rewriting the metabolic code of the host immune system, this strategy treats inflammation not merely as a signaling error but as a thermodynamic imbalance to be corrected.

## 5. Metabolic rescue architectures targeting regulated cell death pathways

Unlike the traditional reductionist target theory, contemporary transcriptomic landscapes reveal that RE is driven by highly immunogenic forms of regulated cell death, specifically ferroptosis and pyroptosis, which are governed not by random genetic hits but by definable, programmable metabolic checkpoints [101].

We propose that radiation-induced intestinal injury constitutes a disease of metabolic determinism. The depletion of critical buffering metabolites, specifically creatine, NAD^+^, and specific indole derivatives, lowers the thermodynamic threshold for these lethal cascades [3],[25],[102]. Consequently, the next frontier of microbial synthetic biology is the design of metabolic rescue chassis: engineered commensals that function as auxiliary metabolic organs to replenish these depleted pools and actively interdict the transition from damage to necrosis [88].

### 5.1. Inhibition of ferroptosis through engineered creatine metabolism

Ferroptosis, an iron-dependent form of cell death driven by lipid peroxidation, is a primary driver of radiation-induced epithelial necrosis [103]. Ionizing radiation upregulates Acyl-CoA synthetase long-chain family member 4, enriching membranes with oxidizable polyunsaturated fatty acids (PUFAs) that serve as fuel for the ferroptotic fire [104]. Emerging evidence suggests that creatine acts as a metabolic brake on this process by activating AMPK, which inhibits fatty acid synthesis and reduces the pool of labile PUFAs available for peroxidation [25].

While recent industrial biotechnology has achieved high-titer creatine production using metabolically engineered *Corynebacterium glutamicum* or *E. coli* in controlled bioreactors, applying this to the human gut remains a significant engineering challenge [88]. The translation gap is substantial: Industrial strains operate under optimized aerobic conditions with abundant precursors, whereas therapeutic probiotics must function in the anaerobic, nutrient-limited environment of the inflamed gut [91]. A viable therapeutic strategy requires refactoring industrial gene clusters, notably *gamt* and *agat*, to function within a commensal chassis like EcN, potentially driven by radiation-responsive promoters to minimize metabolic burden [105].

### 5.2. Modulation of pyroptosis via intracellular NAD^+^ salvage pathways

Radiation-induced DNA damage hyperactivates PARP1, leading to a rapid depletion of the cellular NAD^+^ pool, creating a state of energetic exhaustion that inactivates SIRT1 and unleashes Gasdermin E (GSDME)-mediated pyroptosis [3]. Restoring NAD^+^ levels via its precursor, nicotinamide mononucleotide (NMN), has shown promise in rescuing this phenotype [106],[107]. While enzymatic studies have characterized the kinetics of nicotinamide phosphoribosyltransferase (NAMPT) in synthesizing NMN, transforming this into a living therapeutic faces a critical safety constraint: extracellular NAMPT can act as a pro-inflammatory cytokine [108],[109]. Therefore, a synthetic biology approach prioritizes strict compartmentalization—engineering bacteria to express NAMPT intracellularly and equipping them with specific transporters such as the PnuC exporter to secrete only the beneficial NMN metabolite, thereby decoupling metabolic rescue from immune activation.

### 5.3. Induction of epithelial regeneration utilizing indole-3-carboxaldehyde

The regeneration of the irradiated epithelium relies on *Lgr5*^+^ intestinal stem cells (ISCs). Recent mechanistic discoveries identified indole-3-carboxaldehyde (I3A), a microbial tryptophan metabolite, as a potent radioprotector that induces ISC quiescence and subsequent regeneration via the AhR-IL-10 and AhR-IL-22 axes [102],[110]. However, natural commensals often produce I3A alongside toxic indole. A theoretical indole architect strategy involves metabolic flux redirection: knocking out the tryptophanase gene, *tnaA*, in a *Lactobacillus* chassis to abolish indole production, while overexpressing the aminotransferase pathways to shunt tryptophan exclusively toward I3A biosynthesis [111],[112].

However, the clinical realization of these metabolic architectures faces significant engineering hurdles driven by chassis incompatibility [113],[114]. Industrial pathways from soil bacteria like *Corynebacterium glutamicum* cannot be simply transplanted into gut commensals due to fundamental divergences in codon usage, secretion signals, and regulatory networks. Furthermore, the substantial metabolic burden imposed by high-level metabolite production consumes vital cellular resources, primarily ATP, leading to a fitness cost that risks rapid competitive exclusion by native flora [115]. Consequently, the deployment of these genetically modified organisms requires robust biocontainment measures, such as genetic entanglement, to prevent environmental escape while ensuring therapeutic stability.

While the modulation of creatine, NAD^+^, and indole-3-carboxaldehyde illustrates the potential of metabolic engineering, the synthetic biology toolkit allows for the targeting of a substantially broader spectrum of lethal checkpoints. Beyond these paradigmatic examples, recent efforts have focused on engineering chassis capable of synthesizing complex cofactors and signaling metabolites, such as glutathione, Coenzyme Q10, and ketone bodies, that directly intercept ferroptosis and pyroptosis pathways independent of host machinery. Furthermore, the development of eukaryotic chassis like *Saccharomyces boulardii* has enabled the secretion of polyamines and mitophagy inducers, effectively creating a multi-layered defense against bioenergetic collapse. Key examples of these expanded metabolic rescue strategies, detailing their genetic architectures and therapeutic targets, are presented in Table 4.

**Table 4.** Microbial synthetic biology architectures for metabolic rescue and tissue regeneration.

Metabolite	Microbial chassis	Target mechanism	Synthetic circuit/pathway	Ref
Glutathione	*L. lactis* NZ9000; *E. coli* BL21	Ferroptosis inhibition (GPX4 cofactor)	Bifunctional *gshF* (feedback-resistant) + *metK* (SAM synthase)	[116]
Coenzyme Q10	*E. coli* (Opto-controlled); *C. glutamicum*	Ferroptosis inhibition (FSP1 axis)	*ddsA* (decaprenyl synthase) + *ubiA-J* optimization + optogenetic control	[117],[118]
β-Hydroxybutyrate	EcNL4	Pyroptosis inhibition (NLRP3 blockade)	Reverse β-oxidation (*atoB*, *fadB/phaB*) + Δ*ldhA/adhE/pta*	[119]
Spermidine	*S. boulardii* (Sb576)	Autophagy/mitophagy (eIF5A activation)	*SPE1/2/3* overexpression + *TPO1* transport + Δ*OAZ1*	[120]
Urolithin A	*E. faecium* FUA027; *Enterocloster* spp.	Mitophagy induction (AhR/Nrf2)	*ucd* & *uxd* operons (Molybdenum-dependent dehydroxylases)	[121]
Indole-3-Propionic acid	*E. coli* Nissle; *C. sporogenes*	Barrier integrity (PXR activation)	Reductive Trp pathway (*fldH*, *fldBC*, *acdA*)	[122]

Abbreviations: gshF, bifunctional glutathione synthase; ddsA, decaprenyl diphosphate synthase (CoQ10 biosynthesis); FSP1, ferroptosis suppressor protein 1 (CoQ10 oxidoreductase); atoB/fadB, synthetic β-hydroxybutyrate pathway genes; SPE1/2/3, spermidine biosynthetic genes; TPO1, polyamine transporter; eIF5A, eukaryotic translation initiation factor 5A; ucd/uxd, urolithin A biosynthetic gene clusters; fldH, phenyllactate dehydratase cluster (IPA synthesis).

## 6. Advanced material interfaces and genetic containment for therapeutic robustness

Clinical translation requires reliable delivery hardware and robust biocontainment. Radiation enteritis presents a uniquely hostile engineering environment defined by luminal hyperoxia, oxidative storms, and mucosal stripping. Consequently, the chassis of a living biotherapeutic product must function not merely as a passive carrier but as an active thermodynamic agent capable of surviving the gastric gauntlet while initiating the reconstruction of the anaerobic niche. Representative physical interfaces and genetic strategies, ranging from bio-adhesive coatings to synthetic auxotrophy, are summarized in Table 5.

**Table 5.** Material and genetic engineering strategies for enhanced therapeutic viability and biocontainment.

Category	Platform/strain	Mechanism	Payload/cargo	Ref.
Bio-coating	*B. longum* @ Map/Alg	pH-responsive self-crosslinking shell; adheres to mucosa	Endogenous enzymes/short-chain fatty acids	[123]
Hybrid system	*EcN* + pinocembrin liposomes	Bacteria-liposome conjugate; dual release (colonization + drug)	Pinocembrin (flavonoid)	[124]
Mineralization	*L. casei*/*B. fragilis* @ CaP	In situ mineralization; neutralizes acid; Ca²⁺ sequesters bile acids.	Live bacteria	[125]
Synthetic	Yeast shells + COF-Au	Macrophage-targeted Trojan horse; Au NPs mimic CAT/SOD.	Au nanozymes	[126]
Genetic safety	*EcN* (CRISPR-Cas)	Cellobiose-regulated kill switch; degrades plasmid upon signal loss.	CRISPR machinery	[127]
Auxotrophy	*S. boulardii* (Δ*THI6* Δ*BTS1*)	Dual containment: thiamine auxotrophy + cold sensitivity (<20 °C).	Therapeutic peptides	[128]
Syn. auxotrophy	*E. coli* (orthogonal)	Dependence on synthetic amino acid (BipA); genetic recoding.	N/A (platform)	[62]
Spore ghosts	*Bacillus* spore coats	Non-living shells; passive ROS scavenging; immune modulation.	Antioxidant proteins; PAMPs	[129]
OMV therapy	*EcN* OMVs (ePD-L1)	Mucus-penetrating vesicles displaying anti-PD-L1 antibodies.	Anti-PD-L1; Treg inducers	[130]

Abbreviations: Map, mussel adhesive protein (e.g., Mefp-5); Alg, alginate; CaP, calcium phosphate; COF-Au, covalent organic framework doped with Au nanoparticles (ROS-scavenging nanozymes); OMVs, outer membrane vesicles; ePD-L1, engineered PD-L1 or anti-PD-L1 antibody.

### 6.1. Bio-interfacial engineering of antioxidant armored chassis

Oral delivery of engineered anaerobes faces immediate attrition within the stomach, where acidic pH can reduce viable counts by orders of magnitude [131]. In the irradiated gut, this challenge is compounded by inflammation-induced hypermotility, which drives a rapid washout effect that prevents colonization [2]. Conventional enteric coatings, such as alginate, provide passive shielding but fail to address the active oxidative hostility of the RE microenvironment [18].

To surmount these barriers, we advocate for an armored probiotic strategy utilizing functional bio-interfaces [17]. A paradigmatic advancement in this domain involves the layer-by-layer encapsulation of chassis strains like *Bacillus cereus* or EcN with polydopamine and chitosan [130]. Unlike inert polymers, PDA is rich in catechol moieties that function as potent radical scavengers. By neutralizing luminal hydroxyl radicals and superoxide anions before they penetrate the bacterial cell wall, the PDA armor acts as an exogenous antioxidant enzyme. This activity works in synergy with the engineered metabolic payload to lower the local redox potential and restore the biological oxygen barrier described in the oxygen hypothesis. Simultaneously, the outer chitosan layer confers pH-responsive release and high-affinity mucoadhesion [132]. The positively charged amine groups of chitosan interact electrostatically with the negatively charged mucins on the epithelial surface. This anchoring mechanism resists peristaltic washout and ensures the localized delivery of therapeutic effectors to the site of injury. By integrating prebiotic substrates such as mucin mimetics within this shell, the system further stabilizes the commensal phenotype of candidate strains like *A. muciniphila*, preventing the starvation-induced pathogenicity characterized by the *Akkermansia* paradox.

### 6.2. Biocontainment via genetic entanglement and pseudoessentiality

The deployment of genetically modified organisms in human subjects necessitates fail-safe biocontainment to preclude environmental escape. Traditional kill switches based on toxin-antitoxin systems are prone to evolutionary failure as spontaneous mutations can inactivate the toxin gene and allow escape mutants to proliferate with frequencies as high as 10⁻^5^.

To achieve evolutionary stability, a growing trend in the field favors genetic entanglement, as exemplified by the STALEMATE system [115]. This architecture creates a state of pseudoessentiality by physically overlapping the coding sequence of a biocontainment regulator, such as the immunity protein Im9, with an essential gene or a toxic nuclease. Because the two genes share the same physical DNA sequence read in different frames, they become inextricably linked. Any mutation attempting to inactivate the kill switch to permit escape will simultaneously disrupt the sequence of the essential gene and cause immediate cell death. This topology reduces escape frequencies to negligible levels (<10⁻⁸), a critical threshold for regulatory compliance. Beyond experimental safety, clinical translation faces significant non-experimental barriers. The FDA's guidelines for Live Biotherapeutic Products (LBPs) mandate rigorous environmental risk assessments for genetically modified organisms (GMOs). Systems like STALEMATE are not merely safety features but regulatory prerequisites to satisfy FDA/EMA concerns regarding environmental shedding and horizontal gene transfer [133]. Furthermore, the economic viability of these engineered strains relies on overcoming cold-chain logistical costs to compete with conventional, albeit less effective, pharmacological management. Patient adherence, challenged by the need for frequent dosing of non-colonizing strains, must be addressed through advanced encapsulation or sporulation technologies [115].

### 6.3. Cell-free regenerative therapy using lyophilized apoptotic vesicles

For patients presenting with Grade 3 or 4 toxicity where the mucosal barrier is breached and neutropenia is severe, the administration of live bacteria carries an inherent risk of translocation and sepsis. To resolve this safety paradox, we propose a pivot to cell-free regenerative biologics utilizing lyophilized apoptotic vesicles (Lpl-apoVs).

Distinct from small exosomes, these vesicles are derived from mesenchymal stem cells during apoptosis and are enriched with nuclear repair proteins, including PARP1, alongside functional mitochondrial components. Upon internalization by irradiated epithelial cells, Lpl-apoVs directly repair double-strand DNA breaks and restore mitochondrial bioenergetics via the PINK1-Parkin mitophagy pathway. The capacity to lyophilize these vesicles solves cold-chain logistical challenges and enables the stockpiling of shelf-stable, non-replicating regenerative agents. This approach decouples the metabolic benefits of the holobiont from the risks of microbial replication, offering a precision therapeutic option for the most vulnerable clinical cohorts [134],[135].

## 7. Translational validation frameworks and clinical evolution strategies

**Figure 2.**
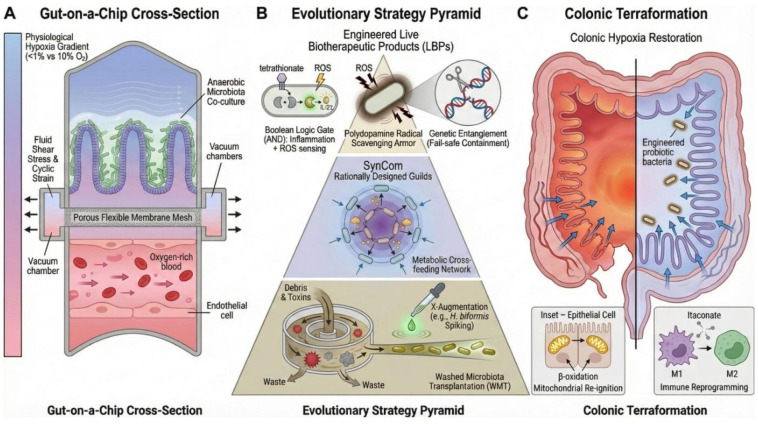
Integrated translational framework for validating and deploying engineered holobiont therapeutics. (A) Microphysiological screening: Utilization of microfluidic gut-on-a-chip systems to model shear stress and anaerobic gradients, alongside patient-derived tumoroids to screen for differential radioprotection. (B) Therapeutic hierarchy: a phased evolution from washed microbiota transplantation (WMT) to synthetic consortia, culminating in logic-gated living biotherapeutic products (LBPs). (C) Colonic terraformation: The clinical endpoint involves active reconstruction of the gut ecosystem, characterized by restored hypoxia, immune tolerance, and metabolic homeostasis.

The clinical translation of microbial synthetic biology is fundamentally constrained by the imperative to decouple epithelial regeneration from tumor preservation [113]. While engineered therapies aim to mitigate intestinal injury, they must undergo rigorous scrutiny to ensure they do not inadvertently confer radioresistance to residual malignancies [136]. Establishing this differential safety profile requires a validation framework that transcends traditional murine surrogates in favor of human-relevant models capable of dissecting tissue-specific responses with high fidelity [137]. To bridge the gap between benchside discovery and bedside application, we propose a multi-layered translational framework. This roadmap integrates advanced microphysiological validation models with a hierarchical evolution of therapeutic strategies, ranging from standardized microbiota transplantation to precision-engineered synthetic circuits (Figure 2).

### 7.1. Microphysiological systems for high-fidelity therapeutic screening

Static cell cultures fail to recapitulate the complex physicochemical forces of the intestine, such as fluid shear stress and the distinct oxygen gradients that define the microbial niche [138]. Therefore, the adoption of microfluidic human gut-on-a-chip systems is advocated as the primary screening modality for engineered strains (Figure 2A). These devices are lined with primary human epithelium and interfaced with microvascular endothelium to allow for the precise modeling of radiation injury in a physiologically active environment. Recent work has validated the predictive power of this platform using an AI-driven drug repurposing algorithm called NemoCAD to identify the antifungal agent Miconazole as a potent radioprotector [105]. The chip revealed that Miconazole functions by inhibiting matrix metalloproteinase-9, which prevents radiation-induced degradation of tight junctions and vascular leakage. This success underscores the necessity of dynamic flow models for validating engineered chassis, specifically for strains engineered to restore barrier function under shear forces.

For personalized stratification, patient-derived organoids and tumoroids offer a critical digital twin capability [139],[140]. By establishing matched organoid lines from a patient's healthy rectal mucosa and their colorectal or pelvic tumor, researchers can conduct differential screening of living biotherapeutic products. A viable candidate strain must meet a strict biological screening criterion where the therapy induces regeneration in normal organoids while exhibiting neutral or cytotoxic effects on tumor organoids [141]. This platform is particularly critical for validating strategies that rely on metabolites like butyrate or indole-3-propionic acid to ensure they do not fuel the metabolic demands of the specific tumor subtype.

The feasibility of decoupling toxicity from efficacy has been definitively demonstrated by the LR-IL-22 strain, a genetically engineered *Limosilactobacillus reuteri* secreting Interleukin-22. In preclinical models of ovarian cancer undergoing whole-abdomen irradiation, oral gavage of LR-IL-22 significantly mitigated intestinal crypt apoptosis and preserved barrier function [142]. Crucially, this intervention did not protect the ovarian tumors but instead reshaped the immune landscape of the tumor microenvironment by recruiting CD8^+^ cytotoxic T cells and upregulating PD-L1 on tumor cells. This effectively transformed cold tumors into hot targets for immune checkpoint blockade and proved that engineered microbes can synergistically enhance the therapeutic index of radiotherapy rather than diminishing it.

### 7.2. Hierarchical strategy for the clinical translation of engineered therapeutics

Clinical translation must navigate significant patient heterogeneity and evolving regulatory frameworks. Consequently, we propose a pyramid of increasing technological sophistication (Figure 2B) [77],[143].

The immediate foundation for clinical translation lies in the refinement of fecal transfer through washed microbiota transplantation (WMT) [24],[144], which has recently demonstrated efficacy in restoring homeostasis across the entire gastrointestinal tract [145]. The replacement of crude fecal microbiota transplantation with automated microfiltration serves to eliminate pro-inflammatory metabolites and planktonic pathobionts. This approach is further refined by X-augmented strategies, exemplified by the spiking of WMT preparations with key functional species such as *H. biformis*. Such augmentation aims to reverse the bioenergetic collapse characteristic of RE. Specifically, *H. biformis*-derived fatty acids activate epithelial PPAR-γ signaling, which reprograms colonocyte metabolism to re-initiate mitochondrial β-oxidation, thereby actively depleting luminal oxygen and restoring physiological hypoxia [24].

As ecological principles are better elucidated, the field advances toward synthetic communities [143]. Moving beyond empirical mixtures, these defined microbial consortia are constructed using bottom-up design principles to ensure ecological stability and metabolic coupling. A primary objective at this stage is the assembly of hypoxia restoration guilds, which are rationally designed networks that synergistically combine oxygen-scavenging facultative anaerobes with obligate anaerobes to actively maintain the anaerobic niche essential for mucosal healing.

The ultimate frontier of this therapeutic evolution is represented by genetically engineered living biotherapeutic products (LBPs). A bridging strategy, WMT, has recently demonstrated efficacy in restoring homeostasis across the entire gastrointestinal tract (small intestine and colon) in animal models [145]. While WMT validates the principle of ecological restoration, logic-gated LBPs represent the theoretical peak of precision medicine, offering targeted control unavailable in undefined consortia [78]. These advanced organisms are designed to feature logic-gated control, restricting therapeutic payload release strictly to the site of injury. The clinical viability of these engineered strains relies on the integration of robust biocontainment systems, such as genetic entanglement (STALEMATE) [115], and sophisticated delivery hardware, such as polydopamine-coated armored chassis, to ensure that efficacy is achieved without compromising biosafety [79],[130].

## 8. Conclusion

Microbial synthetic biology represents a fundamental paradigm shift in the management of radiation enteritis (RE). By embracing colonic terraformation—defined as the active reconstruction of gut metabolic, immunological, and ecological homeostasis—we move beyond symptom management. As demonstrated by the resolution of the *Akkermansia* paradox and the immunological timing paradox, rationally designed engineered microbes achieve precision functionalities unattainable by wild-type strains: delivering therapeutic signals without compromising barrier integrity and autonomously sensing injury phases to modulate immune responses.

**Figure 3.**
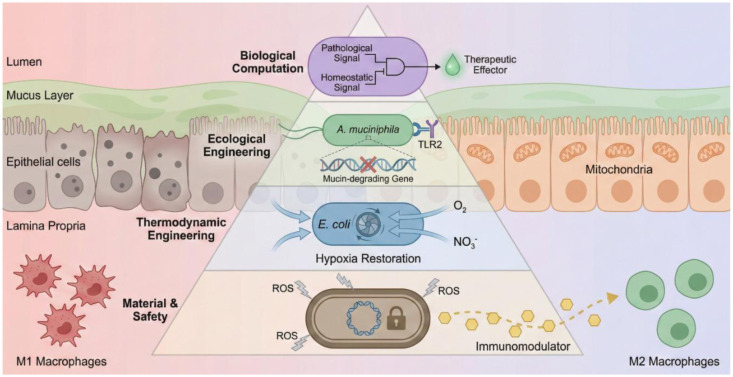
The holobiont engineering pyramid. A hierarchical roadmap for colonic terraformation. Material and safety (bottom layer): Ensures biocontainment via genetic locks and reprograms macrophages (M1-to-M2) for immune tolerance. Thermodynamic engineering (second layer): Restores hypoxia using engineered bacterial oxygen sinks to reverse the oxygen leak. Ecological engineering (third layer): Domesticates *A. muciniphila* by decoupling barrier maintenance (TLR2) from pathogenic mucin degradation. Biological computation (apex): Deploys Boolean logic gates for precise, inflammation-responsive therapeutic release.

The critical challenge ahead lies in integrating these discrete modules into a systemic blueprint for colonic terraformation. We conceptualize this integration as the holobiont engineering pyramid (Figure 3), a hierarchical framework delineating the material, thermodynamic, ecological, and computational layers necessary for restoration. This engineering-driven strategy aims to actively reconstruct the metabolic and immunological architecture of the gut ecosystem (Figure 2C) by incorporating rigorous biocontainment systems, such as STALEMATE, with advanced delivery vehicles. Ultimately, this approach promises to resolve the radiotoxicity paradox—decoupling epithelial regeneration from tumor protection—and expands the definition of cancer survivorship from simple malignancy eradication to the preservation of the holobiont's long-term physiological integrity.

## Use of AI tools declaration

The authors utilized AI-assisted editing tools (DeepSeek) solely for the purpose of improving language fluency and readability. The final content was reviewed and verified by the authors.
